# Expression patterns of CD28 and CTLA‐4 in early, chronic, and untreated rheumatoid arthritis

**DOI:** 10.1002/jcla.23188

**Published:** 2020-01-06

**Authors:** Mariel García‐Chagollán, Iris Yolanda Ledezma‐Lozano, Jorge Hernández‐Bello, Pedro Ernesto Sánchez‐Hernández, Sergio Ramón Gutiérrez‐Ureña, José Francisco Muñoz‐Valle

**Affiliations:** ^1^ Instituto de Investigación en Ciencias Biomédicas Centro Universitario de Ciencias de la Salud Universidad de Guadalajara Guadalajara México; ^2^ Laboratorio de Inmunología Centro Universitario de Ciencias de la Salud Universidad de Guadalajara Guadalajara México; ^3^ Servicio de Reumatología OPD Hospital Civil de Guadalajara “Fray Antonio Alcalde” Guadalajara México

**Keywords:** CD28, CTLA4, Rheumatoid arthritis

## Abstract

**Background:**

T‐cell activation pathways have been proposed as trigger mechanisms in the pathogenesis of rheumatoid arthritis (RA). CD28 and CTLA‐4 play major roles in regulating the stimulatory and inhibitory co‐signals in T cells.

**Objective:**

To analyze the association between soluble and surface expression of CD28 and CTLA‐4 with the clinical parameters of RA patients.

**Methods:**

A total of 35 RA patients classified as early RA (n = 14), chronic RA (n = 14), and untreated RA (n = 7), as well as 7 age‐ and sex‐matched control subjects (CS) were included. Surface expression of CD28 and CTLA‐4 on T cells was evaluated by flow cytometry. Soluble levels of CD28 (sCD28), CTLA‐4 (sCTLA‐4), and anti‐CCP antibodies were measured by ELISA.

**Results:**

A significant lower percentage of CD8 + T cells positive to CD28 (CS = 64.9% vs RA = 42.7%, *P* = .04), and diminished surface expression of CD28 (CS: MFI = 122.9 vs RA: MFI = 33.1, *P* = .006), were found in chronic RA patients compared to CS. Higher sCD28 were observed in early RA patients compared with chronic RA patients (*P* < .05). sCTLA‐4 was found increased in untreated RA patients compared to early RA patients (*P* < .05). sCD28 concentration correlated with anti‐CCP levels (rho = −0.12; *P* = .032). The soluble and surface expressions of CTLA‐4 were not associated with RA clinical parameters.

**Conclusions:**

In RA, the percentage of CD8 + CD28+ T cells decreases and expresses fewer membrane CD28 than CS. sCD28 levels are lower in chronic RA and are associated negatively with anti‐CCP levels. sCTLA 4 levels are lower in early RA patients than in untreated RA patients.

**Abbreviations**
CS: control subjects
RA: rheumatoid arthritis

## 1 INTRODUCTION

Rheumatoid arthritis (RA) is an autoimmune disease characterized by progressive cartilage and bone damage due to persistent joint inflammation where multiple T‐cell activation pathways are involved.^1^, ^2^

Activation of T cells requires at first the binding of the T‐cell receptor (TCR) with a MHC‐peptide complex; however, after triggering of TCR, T‐cell activation is mediated by co‐stimulation signals, which are considered a crucial way to control a T lymphocyte‐mediated immune response and inflammatory reactions.^3^, ^4^

CD28 and the cytotoxic T‐lymphocyte antigen‐4 (CTLA‐4) are the main costimulatory molecules, expressed both by CD4 + T cells and by CD8 + T cells, whose positive and negative signals, respectively, determine the outcome of the T‐cell response to foreign and self‐antigens.^3^, ^5^ CD28 and CTLA‐4 are homologous and belong to the immunoglobulin superfamily, and both molecules interact with the same ligands B7‐1 (CD80) and B7‐2 (CD86) on antigen‐presenting cells (APCs).^3^, ^5^, ^6^ CD28 is constitutively expressed on T cells and delivers an activation signal; on the other hand, CTLA‐4 transduces an inhibitory signal and it is only expressed on activated T cells.^3^, ^5^, ^7^

In RA, the immunopathogenesis is high associated with impaired T‐cell response promoting a proinflammatory microenvironment. Diverse studies have shown the soluble forms of CTLA‐4 in human serum of autoimmune diseases.^8^ Although the biological significance of increased sCTLA‐4 serum levels has not been completely clarified, their possible pathogenetic role during autoimmune disorder can be explained in two ways: sCTLA‐4 inhibits the early T‐cell activation by recognizing CD80/CD86 and blocking the engagement of CD28 expressed on T cells. Conversely, sCTLA‐4 could compete with CTLA‐4 membrane expressed by recognizing CD80/CD86, thus causing a reduction in inhibitory signaling in later T lymphocytes activation phase.^9^, ^10^, ^11^, ^12^, ^13^, ^14^, ^15^

Cao J et al demonstrated higher serum levels of sCTLA‐4 and sCD28 in RA patients than in healthy controls, where serum sCTLA‐4 concentration exhibited a positive and significant correlation with DAS28 score in all RA patients; thus, they proposed that serum levels of sCTLA‐4 could serve as a new marker of RA disease activity; however, they did not evaluate the surface expression of CTLA‐4 and CD28.^16^

Since soluble and membrane expression of CD28 and CTLA‐4 could regulate the outcome of the T‐cell response in RA and contribute to the immunopathogenesis, our purpose was to determine the soluble and membrane expression of CD28 and CTLA‐4 in early, chronic, and untreated RA. Also, we have evaluated the relationship of these molecules with the clinical parameters of the RA patients.

## 2 MATERIALS AND METHODS

### 2.1 Study population

This study included 35 RA patients recruited from the Rheumatology Department at the OPD Hospital Civil Fray Antonio Alcalde, Guadalajara, Jalisco, Mexico. All fulfilled the 2010 American College of Rheumatology/European League Against Rheumatism criteria for RA. These patients were subdivided into three groups: group a) 14 early RA patients (disease duration <2 years); group b) 14 chronic RA patients (disease duration >5 years); and group c) seven untreated RA patients (had not been treated with disease‐modifying anti‐rheumatic drugs, steroids or biological therapy because of their first time in a rheumatology department). At the time of inclusion, the rheumatologist conducted a medical record and evaluation of the disease activity and disability through the Disease Activity Score‐28 (DAS28) and the Health Assessment Questionnaire (Spanish HAQ‐DI), respectively.

The control subjects (CS) group comprised seven unrelated healthy individuals (identified by self‐report) recruited from the general population and matched by sex and age with the RA patients. All individuals were from Western Mexico. This protocol was approved by the Clinical Research Ethics Committee of the University of Guadalajara and Hospital Civil Fray Antonio Alcalde, in Jalisco, Mexico. Informed consent was obtained from all the participants.

### 2.2 Flow cytometry analysis

From peripheral blood samples collected of RA patients and CS, peripheral blood mononuclear cells (PBMC) were obtained by Lymphoprep density‐gradient centrifugation. PBMC were washed with phosphate‐buffered saline supplemented with 0.5% bovine serum albumin. Their viability was evaluated using the trypan blue exclusion method, and only those samples with more than 90% of viability were considered.

Multicolor flow cytometry was used to analyze from PBMC the expression of CD28, CTLA‐4 on gated CD4+, and CD8 + T cells. Cell surface staining was performed with fluorochrome‐labeled monoclonal primary antibodies purchased from eBioscience: FITC anti‐human CD3, PE/Cy5 anti‐human CD8, PE anti‐human CD28, and PE anti‐human CD152. Corresponding isotype control antibodies were also included from eBioscience.

Assay tubes were stained with a mixture of corresponding antibodies at the recommended dilution and incubated for 20 minutes at room temperature in the dark. After incubation, the cells were washed and fixed in PBS containing 1% paraformaldehyde. Finally, data were acquired using EPICS XL‐MCL flow cytometer (Beckman‐Coulter) and cell surface expression of CTLA‐4 and CD28 on CD3CD4 + and CD3CD8 + was analyzed with WinMDI 2.8 software (Scripps Institute, La Jolla, CA).

### 2.3 sCTLA‐4, sCD28, and anti‐CCP antibodies quantification

Serum levels of anti‐CCP antibodies (Axis‐Shield Diagnostics Limited), sCTLA‐4, and sCD28 (Bender Medsystems Diagnostics GmbH) were measured in the RA patients and CS by enzyme‐linked immunosorbent assay (ELISA), according to the manufacturer's instructions. The sensitivities limit of the assays for the sCD28 and sCTLA‐4, and anti‐CCP were 0.18 ng/mL, 0.2 ng/mL, and 1.04 U/mL, respectively. The cutoff level of the anti‐CCP was 5 U/mL for semiquantitative analysis.

### 2.4 Statistical Analysis

Statistical analysis was performed using GraphPad Prism v5.0 software. The Kruskal‐Wallis test was used to analyze differences between three or more groups followed by Dunn's adjustment for multiple comparisons. Spearman's correlation rank test was used to assess the correlations of sCTLA‐4 and sCD28 with clinical parameters. A value of *P* < .05 was considered statistically significant.

## 3 RESULTS

### 3.1 Study population

The demographic, clinical characteristics, and laboratory features of the three RA groups (early RA, chronic RA, and untreated RA) and CS are summarized in Table 1. We observed significant differences in hematologic and inflammatory parameters between groups, where untreated RA patients showed the highest leukocytes (*P* = .0466), platelets (*P* = .0166), and C‐reactive protein (CRP, *P* = .0372) levels. On the other hand, rheumatoid factor (RF; *P* = .0007) and anti‐CCP levels (*P* = .0012) were significantly higher in all three RA groups compared with CS.

**Table 1:** Demographic and clinical characteristics of RA patients and CS

Characteristics	CS (n = 7)	Early RA (n = 14)	Chronic RA (n = 14)	Untreated RA (n = 7)	*P*
Sex (Male/Female)	1/6	0/14	1/13	2/5	.2048
Age (yr)^a^	46.6 ± 17.7	37.1 ± 13.2	56.6 ± 13.6	35.9 ± 8.5	.0040
Disease duration (yr)^a^	NA	0.7 ± 0.2	16.4 ± 9.6	6.2 ± 11.1	<.0001
Leukocytes (mil/mm^3^)^a^	7.4 ± 2.3	7.4 ± 2.0	6.7 ± 1.4	10.1 ± 2.7	.0466
Plaquets (mm^3^)^a^	282.2 ± 79.8	363.6 ± 73.4	298.1 ± 58.0	436.5 ± 132.0	.0166
ESR (mm/h)^a^	15.0 ± 7.6	33.5 ± 16.5	35.1 ± 13.3	28.0 ± 16.6	.1066
CRP (mg/dL)^a^	4.±3.2	8.2 ± 7.3	7.6 ± 5.1	17.9 ± 13.0	.0372
RF (UI/mL)^a^	7.4 ± 0.5	480.6 ± 579.8	163.4 ± 282.8	186.3 ± 228.9	.0007
Anti‐CCP (U/mL)^a^	1.6 ± 0.01	139.4 ± 147.9	125.6 ± 103.6	66.7 ± 114.3	.0012
DAS28^a^	‐	4.3 ± 1.7	4.5 ± 1.7	5.1 ± 0.86	.614
*NSAIDs* b		12 (85.7%)	12 (85.7%)	4 (57.14%)	
Prednisone^b^	‐	4 (28.57%)	0 (0%)	0 (0%)	
*DMARDS* b					
Sulfasalazine^b^	‐	9 (64.28%)	8 (57.14%)	0 (0%)	
Chloroquine^b^	‐	12 (85.7%)	9 (64.28%)	0 (0%)	
Methotrexate^b^	‐	12 (85.7%)	14 (100%)	0 (0%)	

Note

Values are mean ± SD.

Abbreviations: anti‐CCP, anti‐cyclic citrullinated peptide; CRP, C‐reactive protein; CS, control subjects; ESR, erythrocyte sedimentation rate; NA, not applicable; RA, rheumatoid arthritis; RF, rheumatoid factor; yr, year.

^a^ = mean ± SD.

^b^ = n (%).

### 3.2 Decreased expression of CD28 on CD8 + T cells from chronic RA patients

From peripheral blood mononuclear cells, the percentage of total frequency of CD4 + and CD8 + T cells remains unchanged among CS, early RA, chronic RA, and untreated RA patients (*P* > .05, Table 2).

**Table 2:** Expression of CD28 and CTLA‐4 in T cells from RA patients and CS

	CS (n = 7)	Early RA (n = 14)	Chronic RA (n = 14)	Untreated RA (n = 7)	*P*
CD3/CD4%	32.6 (18.0‐57.6)	30.6 (7.2‐47.4)	33.8 (7.3‐46.1)	33.4 (22.7‐44.2)	.753
CD3/CD8%	19.2 (10.4‐23.5)	18.3 (2.2‐28.8)	13.9 (2.2‐25.6)	15.8 (6.8‐22.6)	.831
CTLA4% CD4	0 (0‐0)	0 (0‐1.5)	0 (0‐0.1)	0 (0‐0.3)	.783
CTLA4MFI/CD4	0 (0‐3.9)	0 (0‐14.4)	0 (0‐6.3)	0 (0‐3.6)	.901
CTLA4% CD8	0 (0‐2.2)	0 (0‐51.9)	0 (0‐30.3)	0 (0‐42)	.823
CTLA4MFI/CD8	0.1 (0‐21.6)	0 (0‐39.4)	0.7 (0‐80)	0 (0‐59.3)	.442
CD28% CD4	93.7 (49.1‐98.4)	93.3 (86.9‐96.7)	91 (66.1‐99.2)	95.9 (90.5‐99.5)	.741
CD28MFI/CD4	345.4 (57.4‐764.8)	345.9 (60.0‐701.2)	410.5 (38.3‐1653)	393.3 (123.8‐609.9)	.961
CD28% CD8	64.9 (42.8‐89.7)	58.4 (24.3‐85.9)	42.7 (0.9‐82.6)	60.8 (32.4‐91.0)	.029
CD28MFI/CD8	132.0 (81.1‐212.3)	61.3 (2.9‐319.5)	33.1 (0‐113.2)	61.2 (11.2‐285)	.015

Note

The percentage of T cells positive and cell surface expression (MFI, mean fluorescence intensity) of CTLA‐4 and CD28 on peripheral blood mononuclear cells (PBMC) of RA patients and CS. The differences were determined by Kruskal‐Wallis test. Data are expressed as median (p5‐p95).

Abbreviations: CS, control subjects; RA, rheumatoid arthritis.

The expression of CD28 and CTLA‐4 was analyzed on CD4+ and CD8 + T cells and represented as percentage and MFI. Neither CD4+ nor CD8 + T cells shown cell surface expression of CTLA‐4 in any group of study (Table 2).

**Figure 1:**
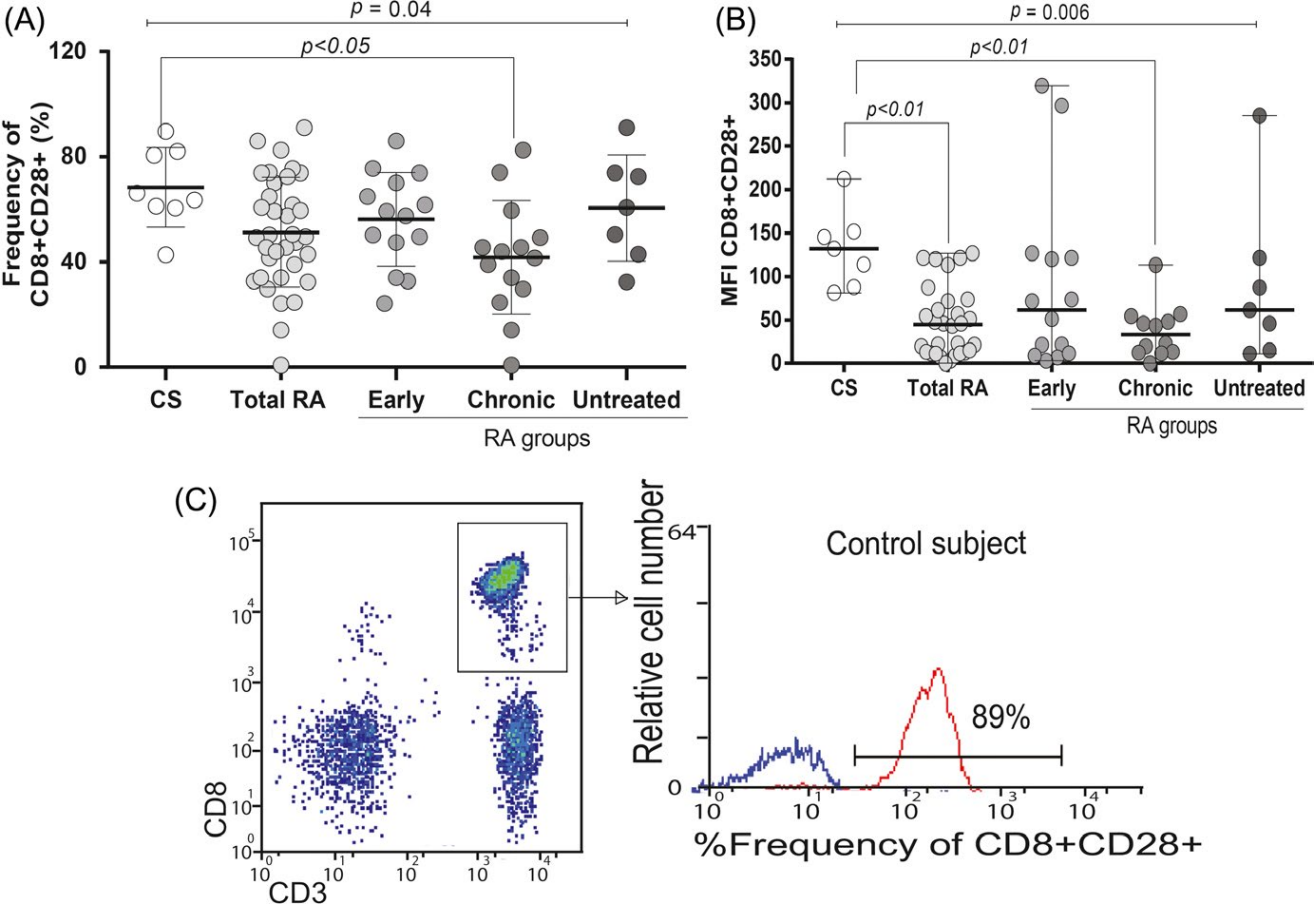
The percentage cell surface expression of CD28 on CD3CD8 + cells of RA patients and CS by flow cytometry. A, Reduced percentage and (B) depicted MFI expression of CD28 on CD3CD8 + cells in chronic RA patients. The data were analyzed by the Kruskal‐Wallis test. C, A representative example is shown, at first lymphocyte populations were gated according to their forward scatter (FS) and side scatters (SS) characteristics. Further, cells from the lymphocyte gate were subgated based on CD3 expression and CD8 co‐expression. Histogram reflects the portion of CD28 expression within the given CD8 + T‐cell populations

On the other hand, the membrane CD28 expression was found in both CD4+ and CD8 + T cells in all groups; however, no differences were found in CD28 expression on CD4 + T cells (*P* > .05). In contrast, we observed a reduction in the frequency of CD3 + CD8 + CD28 + T cells from RA patients compared with CS (*P* = .029, Table 2); interestingly, once total RA patients group was divided in early, chronic, and untreated, we observed that the most significant decrease in the frequency of these cells was between the group of chronic patients compared to the CS (CD3 + CD8 + CD28 + T cells %: chronic RA = 42.7 vs CS = 64.9, *P* < .05; Figure 1A). Moreover, MFI expression of CD28 on CD8 + T cells also decreased in patients compared to CS (*P* = .015, Table 2), with a marked difference between chronic RA patients and CS (median MFI = 33.1 vs median MFI = 132, respectively, *P* < .01; Figure 1B). A representative example is depicted at Figure 1C where lymphocytes were subgated based on CD3 expression and CD8 co‐expression. CD28 expression within the given CD8 + T‐cell populations is shown in a histogram.

### 3.3 Soluble levels of sCTLA‐4 and sCD28

**Figure 2:**
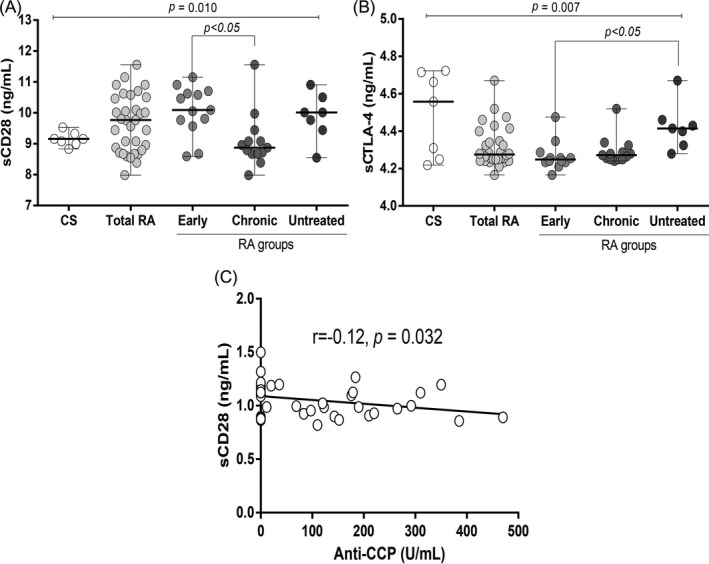
sCD28 and sCTLA4 levels. A, and (B) Serum concentration of sCD28 and SCTLA4 in RA patients and CS. The differences between RA patients and CS were determined by Kruskal‐Wallis test. Data are expressed as median (p5‐p95). C, Correlation between sCD28 and anti‐CCP in 35 RA patients. Spearman's test was used as the statistical test

As shown in Figure 2A, serum concentrations of sCD28 are different between RA patients groups compared to CS (*P* = .010), with a tendency of higher sCD28 levels in RA. On the other hand, the analysis within the RA groups showed sCD28 levels significantly lower in chronic RA patients compared with early RA patients (8.8 ng/mL [7.9‐11.1] vs 10.1 ng/mL [8.5‐11.1], respectively, *P* < .05). Moreover, sCD28 correlated with anti‐CCP antibodies levels (rho = −0.12, *P* = .032; Figure 2C).

Regarding to sCTLA4 levels, we found differences between CS and RA patients (*P* = .007), with a tendency of lower sCTLA4 levels in the RA patients. Interestingly, when dividing RA patients’ groups, early RA patients shown significantly lower levels compared to those untreated (4.2 ng/mL [4.1‐4.4] vs 4.4 ng/mL [4.3‐4.7], respectively, *P* < .05, Figure 2B). Concentrations of sCTLA‐4 did not correlate with any laboratory parameters (*P* > .05, data not shown).

## 4 DISCUSSION

RA patients may exhibit a variety of hematologic and inflammatory abnormalities; in the active disease, this manifestations can include anemia of chronic disease, thrombocytosis, a mild leukocytosis, and high levels of acute‐phase reactants.^17^ However, it is also well known that some DMARDs as methotrexate can counteract these effects and the same time be associated with cytopenia, including leukopenia and thrombocytopenia. In this respect, we observed highest leukocytes, platelets, and C‐reactive protein levels in those untreated RA patients, which could be a result of the lack of treatment.^18^ On the other hand, we observed the presence of seropositivity to autoantibodies (RF and anti‐CCP) in the three RA groups analyzed (early, chronic, and untreated), but not in the CS, which denotes the specificity and clinical importance of these markers from early stages of the disease.

T cells are an important target for immunotherapy in different autoimmune diseases due to an aberrant expression of their co‐stimulation molecules that leads to the loss of self‐tolerance.^12^ The importance of the costimulatory molecules CD28 and CTLA‐4 in the pathologic mechanism of RA has been demonstrated by genetic associations and the successful clinical application of CTLA‐4Ig for the treatment of RA.^19^

In the present study, membrane expression of CTLA‐4 on CD4+ and CD8 + T cells was not found in any group of the study, but all groups had detectable soluble CTLA‐4 (sCTLA‐4) in serum. Similar to us, Pistillo et al also reported a lack of CTLA‐4 expression on the surface, in freshly isolated PBMCs, T cells, B cells, CD34 (+) stem cells, and granulocytes of healthy subjects, but they observed a high expression within the cytoplasm after various treatments with agents able to specifically activate each cell type.^20^

Regarding RA, very low levels of intracellular CTLA‐4 expression in T cells of RA patients, and this expression is lower than in CS, were reported.^14^ This finding suggests a defected membrane CTLA‐4 expression in T cells from RA patients, which has been reported in regulatory T cells and associated with autoimmunity.^21^

On the other hand, sCTLA‐4 is generated by alternatively spliced mRNA and is constitutively expressed on non‐stimulated T cells, while the membrane expression of CTLA‐4 is associated only in activated T cells.^13^, ^15^, ^22^ It has been shown that sCTLA‐4 possesses B7 binding activity and interferes with the interaction between B7 expressed by APC and CTLA‐4 expressed on T cells.^13^, ^15^, ^22^

An increase of sCTLA‐4 has been reported by AlFadhli in the serum of RA patients^23^; in contrast, in this study, we found a trend of lower sCTLA‐4 levels in the total of RA patients in comparison with the CS group (*P* = .09). This discrepancy could be due to the disease activity or the treatment of the RA patients in both study groups; however, AlFadhli did not show the clinical data of their RA patients, so this should be clarified in future studies. It is possible that the lower sCTLA‐4 levels observed in our study can be associated with the treatment of the patients, as a higher level of sCTLA‐4 was observed in those untreated RA patients. Since RA pathogenesis is mediated mainly by T cells, it is possible that if CTLA‐4 plays a role in the pathogenesis of RA, it is mediated by the blocking of B7 leading to T‐cell hyperactivation and autoreactivity.

CD28 is a molecule constitutively expressed at the cell surface; thus, the presence of T lymphocytes subsets deficiency in CD28 expression is associated with autoimmune diseases, such as multiple sclerosis, type 1 diabetes, Graves’ disease, ankylosing spondylitis, and RA.^24^ We found almost 100% of the CD4 + T cells expressing CD28 in RA patients and CS, but no differences were observed among groups. This contrasts the other reported studies an expanded subset of CD4 + T cells characterized by a deficiency of CD28 expression (CD28null) and autoreactive behavior in RA patients^25^, ^26^; therefore, more studies are needed to clarify these findings.

In our study, a significant reduction in the frequency of CD3 + CD8 + T cells positive for CD28 from chronic RA patients compared with CS was observed. Unlike the cytotoxic role exerted by CD4 + CD28 null T cells described in RA, a regulatory behavior of CD8 + CD28 − T cells has been identified in infections, cancer, and autoimmune diseases.^27^ These lymphocytes are able to inhibit a Th1‐type response,^28^, ^29^, ^30^ which has a pivotal role to maintain a proinflammatory microenvironment in RA.

CD8 + CD28 − T cells with immunosuppressive role might be favorable to RA disease to control the severity of the inflammatory response; however, a subset of these cells with defects in its regulatory suppressor function in RA patients has been demonstrated.^28^ In our findings, we observed no changes in the total percentages of CD8 + T cells among RA groups and CS; thus, the diminished frequency and expression of CD8 + T cells positive to CD28 could reflect an inversely proportional increase of CD8 + T cells negative for CD28 in chronic RA patients.

It has been proposed that CD28 absence in T lymphocytes is associated with two biological events: cell senescence^31^, ^32^ and extended exposure to antigens.^33^ However, there are controversial studies showing that there is no correlation between aging and the expression levels of CD8 + CD28 − T‐cell population.^34^ In this respect, we do not find a significant correlation between the CD3 + CD8 + T cells positive for CD28 and the age of RA patients (data not shown). According to some models for suppression, CD8 + CD28 − T cells mediate their functions through the secretion of suppressive cytokines IL‐10 and TGF‐β, so they are considered as regulator cells^35^ and could be produced as an attempt to counteract the inflammatory response that characterizes RA.

Interestingly, a reduced membrane expression (MFI) of CD28 on CD8 T cells from the total RA patients compared to CS was found. This decrease was more prominent between chronic RA patients and CS. This finding could be reflecting the long‐time treatment of the chronic RA patients, as it has been reported that some anti‐rheumatic drugs such as to abatacept are associated with a concomitant decrease in CD8 + CD28 − T cells.^24^ However, we have not found significant differences between RA patients under treatment compared to those untreated. These results may imply that the CD8 + CD28 − T‐cell population is not related to the treatment, but they could be already present since the beginning of the disease.

Other important findings of this study were a trend of higher sCD28 levels in the RA patient groups compared with CS, and a significantly decrease in these levels in chronic RA patients compared with early RA patients. These findings are related to those previously reported in the study.^36^ High levels of sCD28 in RA patients could suggest a regulatory mechanism to compensate the activation in T cells. Interestingly, in our study, an increase of sCD28 correlated with decreased levels of anti‐CCP antibodies. This correlation could be explained due to sCD28 levels could act as an inhibitory molecule, preventing the interaction between CD28 on the surface membrane and their receptors, which represent an important component of the functional interactions between T and B cells.

## 5 CONCLUSIONS

We have provided evidence that CD8 + CD28 + T cells percentage is decreased in RA patients and these cells express membrane CD28 lower than CS. Moreover, sCD28 levels are lower in patients with chronic RA and are associated negatively with anti‐CCP levels. On the other hand, sCTLA4 levels are increased in untreated RA patients but are diminished in patients in early RA patients, maybe after the treatment. However, more studies related to treatment are required to elucidate this association.

One of the limitations of this study is the small number of samples included especially in the group of healthy controls, so these findings should be interpreted with caution and as preliminary results. Future studies in a larger number of samples are needed to investigate the role of the CD28null T cells in disease mechanism and elucidate whether they contribute to the autoantibodies production in RA patients.

## CONFLICT OF INTEREST

The author(s) declare that they have no conflict of interests.

## Data Availability

The data used to support the findings of this study are included within the article.
